# Quantifying the Entropy of Binding for Water Molecules in Protein Cavities by Computing Correlations

**DOI:** 10.1016/j.bpj.2014.12.035

**Published:** 2015-02-17

**Authors:** David J. Huggins

**Affiliations:** 1 Theory of Condensed Matter Group, Cavendish Laboratory, University of Cambridge, Cambridge, UK

## Abstract

Protein structural analysis demonstrates that water molecules are commonly found in the internal cavities of proteins. Analysis of experimental data on the entropies of inorganic crystals suggests that the entropic cost of transferring such a water molecule to a protein cavity will not typically be greater than 7.0 cal/mol/K per water molecule, corresponding to a contribution of approximately +2.0 kcal/mol to the free energy. In this study, we employ the statistical mechanical method of inhomogeneous fluid solvation theory to quantify the enthalpic and entropic contributions of individual water molecules in 19 protein cavities across five different proteins. We utilize information theory to develop a rigorous estimate of the total two-particle entropy, yielding a complete framework to calculate hydration free energies. We show that predictions from inhomogeneous fluid solvation theory are in excellent agreement with predictions from free energy perturbation (FEP) and that these predictions are consistent with experimental estimates. However, the results suggest that water molecules in protein cavities containing charged residues may be subject to entropy changes that contribute more than +2.0 kcal/mol to the free energy. In all cases, these unfavorable entropy changes are predicted to be dominated by highly favorable enthalpy changes. These findings are relevant to the study of bridging water molecules at protein-protein interfaces as well as in complexes with cognate ligands and small-molecule inhibitors.

## Introduction

Experimental techniques such as x-ray crystallography commonly identify water molecules in the internal cavities of proteins (1,2). A number of analyses on databases of protein structures have shown that this is a common observation and that protein cavities commonly accommodate one to three water molecules (3,4). Such water molecules tend to be stabilized by hydrogen bonding interactions and are thus expected to exhibit strong translational and orientational ordering (5). Numerous computational studies have been performed to quantify the binding free energy of such water molecules using free energy methods (6–9). In this context, the binding free energy is the free energy of transfer of a water molecule from the bulk to the cavity. As expected, the binding free energies for crystallographically observed water molecules in protein cavities are predicted to be favorable. However, the degree of ordering relative to bulk water may mean that this favorable free energy is comprised of a favorable enthalpy component and an unfavorable entropy component (5). Analysis from Dunitz calculated the entropy contribution of individual water molecules to the entropy of inorganic crystals (10). This analysis suggests that an entropic cost of transferring an individual water molecule to a protein cavity will not typically be greater than 7.0 cal/mol/K. This corresponds to a contribution of approximately +2.0 kcal/mol to the free energy. From this data, the conclusion was that water molecules in proteins are unlikely to make an entropic contribution of greater than +2.0 kcal/mol to the free energy. In this study, we employ the statistical mechanical method of inhomogeneous fluid solvation theory (IFST) (5,11–14) to quantify the enthalpic and entropic contributions of individual water molecules in protein cavities. IFST has previously been used to rationalize kinase selectivity (15), identify ligand-binding hotspots at protein surfaces (16), and understand the hydrophobic effect (17). It has proven a very useful tool in modeling networks of water molecules in biological systems. IFST has also been used to study water molecules in cavities of IL-1β (18), and in this article we extend such a study to five different proteins and nineteen protein cavities. In addition, we combine the k-nearest neighbors (KNN) (19,20) algorithm with information theory (21–24) to study doubly occupied cavities and explicitly calculate the contributions of changes in water-water correlations and the associated entropy changes. To our knowledge, this approach represents the first estimate of this quantity using mutual information and provides a complete framework to calculate two-particle entropies, as envisioned in the original development of IFST (11,12).

## Materials and Methods

Five test systems were chosen for this study: IL-1β (2), T4 Lysozyme (1), FKBP-2, Carbonic Anhydrase (CA-II) (25), and β-Lactamase (26) (see Table 1). From these five structures, 19 internal cavities were identified. Fifteen of these cavities were singly occupied and four were doubly occupied, and thus the analysis considers 23 water molecules in total. Details of the PDB crystal structures and the residue numbers for these 23 water molecules are listed in Table 1.

### System setup

Protein structures were downloaded from the Protein Databank (27). Selenomethionines were changed to methionines and missing sidechains were added using Schrodinger’s Preparation Wizard (28), which was also used to check the orientations of the asparagine, glutamine, and histidine residues, as well as the protonation state of all ionizable residues. All heteroatomic species such as buffer solvents and ions were removed, with the exception of the zinc ion in the case of Carbonic Anhydrase. The changes made to each structure to improve the hydrogen bonding patterns are detailed in Table 1. The hydrogen-atom positions were then built using the HBUILD facility of CHARMM (29) with the CHARMM27 energy function (30,31), and the force field parameters and partial charges were assigned from the CHARMM27 force field (30,31). Only the crystallographic water molecules identified in internal cavities were retained, with all others being deleted. Water molecules were modeled with the TIP4P-2005 water model (32). To ensure an overall charge of zero in the system, nine and one additional chloride ions were added in the cases of T4 Lysozyme and Beta-Lactamase respectively. All ions were placed farther than 15 Å from the protein.

### Molecular dynamics simulations

Equilibration was performed for 1.0 ns in an NVT ensemble at 300 K using Langevin temperature control (33). All systems were brought to equilibrium before continuing, by verifying that the energy fluctuations were stable. MD simulations were performed using an MD time step of 2.0 fs. Electrostatic interactions were modeled with a uniform dielectric and a dielectric constant of 1.0 throughout the equilibration and production runs. Van der Waals interactions were truncated at 11.0 Å with switching from 9.0 Å. Electrostatics were modeled using the particle mesh Ewald method (34), and the systems were treated using rhombic dodecahedral periodic boundary conditions. All non-water atoms were fixed for the entirety of the equilibration and production simulations. MD simulations were performed using NAMD version 2.9 (35). To explore the effect of protein conformation on the IFST results, we generated 10 conformations of T4 Lysozyme by running a 10.0 ns simulation with heavy-atom restraints of 1.0 kcal/mol/Å^2^ and storing the coordinates every 1.0 ns. Each unique protein conformation was then used for a separate 10.0 ns IFST calculation with a fixed protein structure. For comparison, the 10.0 ns simulation with heavy-atom restraints was also analyzed.

### Free energy perturbation calculations

Free energy perturbation (FEP) calculations were performed for each internal cavity, to calculate the binding energy of the buried water molecules (Δ*G*_*bind*_). The total free energy change for an FEP calculation (Δ*G*_*FEP*_) was calculated as the sum of free energy changes for a series of N small steps between intermediate states a and b (36). The change in free energy was calculated for each small step (Δ*G*_*a*→*b*_) using the partition functions (*Q*) for the two states, which are calculated from the Hamiltonians (*H*):

(1) $$\Delta G_{FEP}=\sum_{a=1,\;b=a+1}^{N}\Delta G_{a\to b}\;$$

(2) $$\begin{array}{c} \Delta G_{a\to b}=G_{b}-G_{a}\\ =-kT\;\ln\left(\frac{Q_{b}}{Q_{a}}\right)\\ =-kT\;\ln\left({\langle \text{exp}\left(-\left(H_{b}-H_{a}\right)/kT\right)\rangle }_{a}\right) \end{array}.$$

**Figure 1:**
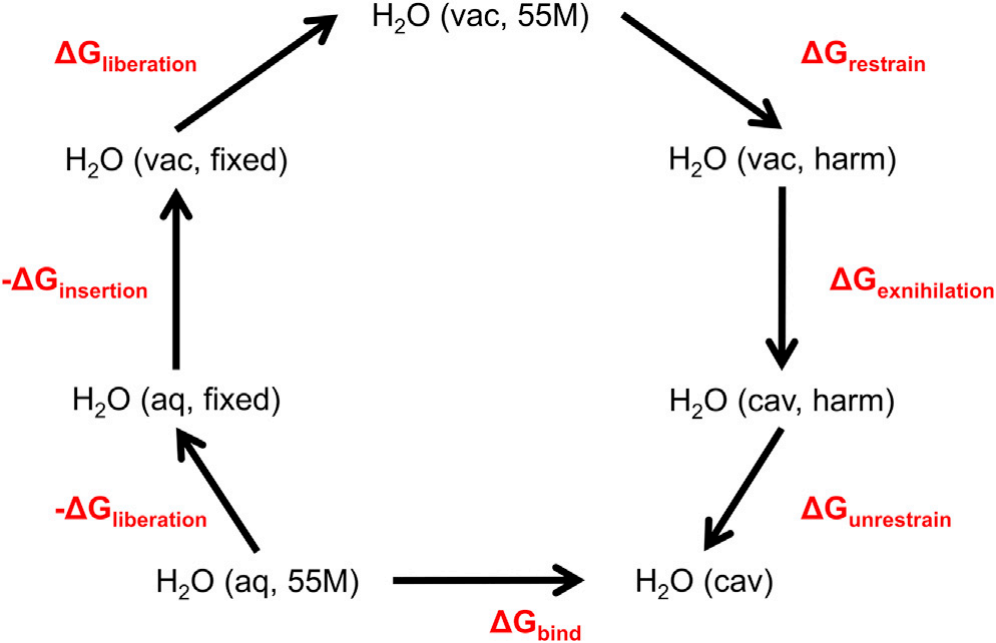
The steps in the free energy cycle used to calculate ΔG_bind_ for a water molecule. The term aq represents a water molecule in aqueous conditions, the term vac represents a water molecule in vacuum, and the term cav represents a water molecule in a protein cavity. 55 M represents a water molecule at the standard bulk concentration of 55 mol/dm^3^, fixed represents a water molecule that is at a fixed point, and harm represents a water molecule that is harmonically restrained. To see this figure in color, go online.

The results for the forward and backward FEP simulations were combined using the Bennett Acceptance Ratio (BAR) method (37,38). BAR was implemented using the ParseFEP Plugin from Visual Molecular Dynamics and the statistical error was estimated in each case (39). The estimated statistical error in the FEP free energy predictions using BAR was less than 0.1 kcal/mol in all cases. We used 32 equally spaced λ windows for the forward FEP simulations and 32 equally spaced λ windows for the backward FEP simulations. A soft-core potential was employed with a van der Waals radius-shifting coefficient of 5.0 (40,41), electrostatic interactions were scaled down to zero between λ = 0.0 and λ = 0.5, and van der Waals interactions were scaled down to zero between λ = 0.5 and λ = 1.0 (38). Equilibration was performed for 250 ps for each lambda window, and production simulation was performed for 750 ps for each lambda window. An NVT ensemble was used throughout and all nonwater atoms were fixed for the entirety of the FEP simulations. The free energy cycle for calculating Δ*G*_*bind*_ can be seen in Fig. 1. The first step transfers the water molecule from 55 M to a fixed point in solution (−Δ*G*_*liberation*_), and the second step annihilates the water molecule from bulk (−Δ*G*_*insertion*_). Δ*G*_*insertion*_ was calculated to be −6.95 kcal/mol for a fixed TIP4P-2005 water molecule using FEP at 1 atm and 300 K in an NPT ensemble. The third step transfers the water molecule from the fixed location back to 55 M in vacuum (Δ*G*_*liberation*_), and thus the two liberation terms in the cycle cancel one another. The fourth step is to harmonically restrain the oxygen atom of the water molecule to aid convergence of the free energy calculations (7,9,42,43). This harmonic restraint leads to an analytic free energy penalty (Δ*G*_*restrain*_) given by Eq. 3:

(3) $$\Delta G_{restrain}=-RT\;\ln\left[C_{0}V_{harm}\right]$$

(4) Vharm=(2πRTkharm)32,

where *C*_0_ is the concentration of a water molecule in bulk (55 M), *V*_*harm*_ is the volume available to the harmonically restrained water molecule, and *k*_*harm*_ is the harmonic force constant. The value of *k*_*harm*_ was set to 0.5 kcal/mol/Å^2^ in all cases, and thus Δ*G*_*restrain*_ was calculated to be +0.23 kcal/mol. The fifth step is exnihilation of the water molecule in the cavity (Δ*G*_*exnihilation*_) using FEP. The final step is to remove the harmonic restraint (Δ*G*_*unrestrain*_) and this contribution is assumed to be zero, as in previous work, and is justified because the dynamics of the water molecule in the cavity are not affected by a force constant that is small in relation to the atomic fluctuations (9,18). For cavities containing two water molecules, the exnihilation is performed on both simultaneously and interactions between the exnihilated water molecules are also scaled to decouple them (18). Considering the steps described above, Δ*G*_*bind*_ can be calculated using Eq. 5:

(5) $$\Delta G_{bind}=\Delta G_{exnihilation\;}-\Delta G_{insertion}+\Delta G_{restrain}.$$

The symmetry contribution to the binding free energy (−0.41 kcal/mol in the case of a water molecule) is only appropriate if there is a difference between the sampling of the symmetry-related states in the bound and unbound states (44). The unbound water molecules are treated as fixed and cannot sample the two symmetry-related states. In this case, the bound water molecules were not observed to sample the two symmetry-related states, presumably because of a large kinetic barrier. Thus, there is no symmetry contribution.

### IFST calculations

IFST calculates the hydration free energy of a solute (Δ*G*_*IFST*_) by computing the difference in free energy between a solution and the same number of solvent molecules (*n*) modeled as the pure bulk solvent (11,12). Δ*G*_*IFST*_ can be calculated for small subvolumes of the system, allowing the contribution of specific regions to be estimated (13,45,46). In the context of protein systems, water molecules tend to cluster at distinct locations termed hydration sites, and it is natural to compute the contribution of individual water molecules to the hydration free energy (14,18,47,48). For the IFST calculations, 100.0 ns of production simulation in an NVT ensemble were performed at 300 K for each system. System snapshots were saved every 5.0 ps, yielding 20,000 snapshots in total for each system. We calculated a mean and standard deviation for each of the IFST quantities by considering 10 blocks from the 100 ns simulation, each derived from 2000 randomly selected snapshots. Δ*G*_*IFST*_ is calculated from the contributions of the hydration energy (Δ*E*_*IFST*_) and hydration entropy (Δ*S*_*IFST*_) to the hydration free energy:

(6) $$\Delta G_{IFST}=\Delta E_{IFST}-T\Delta S_{IFST}.$$

Δ*E*_*IFST*_ is calculated from the mean solute-water interaction energy (*E*_*sw*_), the mean water-water interaction energy (*E*_*ww*_), and the mean interaction energy of a bulk water molecule (*E*_*bulk*_):

(7) $$\begin{array}{c} \Delta E_{IFST}=E_{sw}+E_{ww}-nE_{bulk}\\ =E_{sw}+\Delta E_{ww} \end{array}.$$

*E*_*bulk*_ and *E*_*ww*_ are defined as half the interaction energy of a water molecule with all other water molecules in the system. For the TIP4P-2005 water model, *E*_*bulk*_ is calculated to be −11.5748 kcal/mol. The total hydration entropy is calculated as the sum of local (*S*_*local*_) and nonlocal contributions (*S*_*nonlocal*_). *S*_*local*_ is the summed contribution of each local subvolume and *S*_*nonlocal*_ is the sum of the volume entropy (*S*_*ve*_) and the change in liberation entropy (Δ*S*_*lib*_) (12). *S*_*local*_ is calculated from the two-particle correlation function (*g*_*sw*_) that is a function of the position (*r*) and orientation (ω) of the water molecule. The number density of bulk TIP4P-2005 water (ρ) is calculated to be 0.03324 molecules/Å^3^:

(8) $$\begin{array}{c} \Delta S_{local}=-R\rho \int \left[g_{sw}\;\ln\;g_{sw}-g_{sw}+1\right]dV+\Delta S_{ww}\\ =-R\rho \int g_{sw}\;\ln\;g_{sw}dV-R\rho \int \left[1-g_{sw}\right]dV+\Delta S_{ww}\\ =\Delta S_{IFST}-R\rho \int \left[1-g_{sw}\right]dV \end{array}$$

(9) $$\begin{array}{c} \Delta S_{nonlocal}=S_{ve}+\Delta S_{lib}\\ =-R\left(1-\rho V_{s}\right)+R\left(\alpha T-\rho \kappa kT\right)\\ =-R+R\rho V_{s}+R\alpha T-R\rho \kappa kT\\ \begin{array}{l} =-R+R\rho \left(\kappa kT+\int \left[1-g_{sw}\right]dV\right)+R\alpha T-R\rho \kappa kT\\ =R\left(\alpha T-1\right)+R\rho \int \left[1-g_{sw}\right]dV \end{array} \end{array}$$

(10) $$\begin{array}{c} \Delta S_{hydration}=S_{local}+S_{nonlocal}\\ =\Delta S_{IFST}+R\left(\alpha T-1\right) \end{array},$$

$$\approx \Delta S_{IFST}-R$$

where *V*_*s*_ is the partial molar volume of the solute, α is the thermal expansion coefficient of the solvent, and κ is the isothermal compressibility of the solvent. Equation 9 uses the Kirkwood-Buff relationship for *V*_*s*_ (49). The two remaining integrals in Eqs. 8 and 9 can be understood as a local term corresponding to a reduction in the volume accessible to the solvent (*V*_*s*_*)* and a non-local term corresponding to an increase in the volume accessible to the solvent (*V*_*s*_) as the system expands under constant pressure. These two terms are equal and cancel one another. Δ*S*_*IFST*_ is calculated from the two-particle correlations using the solute-water entropy (*S*_*sw*_) and the difference in water-water entropy (Δ*S*_*ww*_); higher-order correlations are not considered:

(11) $$\begin{array}{c} \Delta S_{IFST}=S_{sw}+S_{ww}-nS_{bulk}\\ =S_{sw}+\Delta S_{ww} \end{array}.$$

For the TIP4P-2005 water model, *S*_*bulk*_ is calculated from the values for *E*_*bulk*_ and Δ*G*_*insertion*_ to be -15.5097 cal/K/mol. In recent work, we developed a novel KNN approach to calculate *S*_*sw*_ using the translational and orientational distance metrics (50). The translational distance (*d*_*trans*_) between two water molecules is simply the Euclidean norm between the Cartesian coordinates of the two water molecules. The orientational distance (*d*_*orient*_) between two water molecules is the distance between the rotations required to bring the two orientations to the same reference orientation. The correct distance metric for the rotation group is twice the geodesic distance on the unit sphere (51). The KNN algorithm provides an unbiased estimate of the absolute entropy (*H*_*abs*_) from the general expression in Eq. 12:

(12) $$H_{abs}=\frac{1}{n}\sum_{i=1}^{n}ln\left[\frac{nd_{i,k}^{p}\pi^{p/2}}{\text{Γ}\left(p/2+1\right)}\right]-L_{k-1}+\gamma$$

(13) $$L_{j}=\sum_{i=1}^{j}\frac{1}{i},$$

where *n* is the number of samples, *d*_*i*,*k*_ is the distance between sample point *i* and its kth nearest neighbor, *p* is the number of degrees of freedom, Г is the gamma function, *L*_0_ is 0, and γ is Euler’s constant. In the context of an individual water molecule, the relative solute-water thermodynamic entropy (*S*_*sw*_) is calculated using the total distance (*d*_*total*_) between two water molecules in six dimensions (*p* = 6) (50). It is calculated from the difference between the absolute entropy of the distribution (*H*_*abs*_) and the absolute entropy of a uniform distribution (*H*_*uni*_). We disregard the symmetry number (two in this case) as it is present in both *H*_*abs*_ and *H*_*uni*_. We use the first nearest neighbor in this work (*k* = 1) and the number of samples is equal to the number of frames where a water molecule is present in the hydration site:

(14) $$\begin{array}{c} S_{sw}=R\left\{H_{abs}-H_{uni}\right\}\\ =R\left\{\frac{1}{n}\sum_{i=1}^{n}\ln\left[\frac{nd_{total}^{6}\pi^{3}}{\text{Γ}\left(4\right)}\right]-L_{0}+\gamma -\ln\left[\frac{8\pi^{2}}{\rho }\right]\right\}\\ =R\left\{\frac{1}{n}\sum_{i=1}^{n}\ln\left[\frac{nd_{total}^{6}\pi^{3}}{6}\right]+\gamma -\frac{1}{n}\sum_{i=1}^{n}\ln\left[\frac{8\pi^{2}}{\rho }\right]\right\}\\ =R\left\{\frac{1}{n}\sum_{i=1}^{n}\ln\left[\frac{nd_{total}^{6}\pi \rho }{48}\right]+\gamma \right\} \end{array}$$

(15) $$d_{total}=\sqrt{d_{trans}^{2}+d_{orient}^{2}},$$

(16) $$d_{trans}=\sqrt{{\left(x_{ij}-x_{kl}\right)}^{2}+{\left(y_{ij}-y_{kl}\right)}^{2}+{\left(z_{ij}-z_{kl}\right)}^{2}},$$

(17) $$d_{orient}=2\times \text{acos}\left(\left|{\text{q}}_{\text{ij}}.{\text{q}}_{\text{kl}}\right|\right),$$

where *R* is the gas constant and converts the entropy to thermodynamic units and Г(4) is equal to 6. The Cartesian coordinates of water molecule *j* in frame *i* and its nearest neighbor water molecule *k* in frame *l* are denoted by *x*_*ij*_, *y*_*ij*_, *z*_*ij*_ and *x*_*kl*_, *y*_*kl*_, *z*_*kl*_, respectively. The quaternion representations of the rotations for water molecule *j* in frame *i* and its nearest neighbor water molecule *k* in frame *l* are denoted by *q*_*ij*_ and *q*_*kl*_, respectively. In this work, we extend the nearest neighbor approach to calculate *S*_*ww*_ from the two-particle correlation functions (*g*_*sw*_, *g*_*sw*'_, and *g*_*ww*'_) and the triplet correlation function (*g*_*sww*'_), which is a function of the 12 variables representing the positions and orientations of a pair of water molecules (*p* = 12):

(18) $$\begin{array}{c} S_{ww}=-\frac{1}{2}R\rho^{2}\iint g_{sw}g_{sw^{\prime }}\left[g_{ww^{\prime }}\;\ln\;g_{ww^{\prime }}-g_{ww^{\prime }}+1\right]dwdw^{\prime }\\ =-\frac{1}{2}R\rho^{2}\iint g_{sw}g_{sw^{\prime }}g_{ww^{\prime }}\;\ln\;g_{ww^{\prime }}dwdw^{\prime }-\frac{1}{2}R\rho^{2}\iint g_{sw}g_{sw^{\prime }}\left[1-g_{ww}\right]dwdw^{\prime }\\ =-\frac{1}{2}R\rho^{2}\iint g_{sww^{\prime }}\;\ln\left[\frac{g_{sww^{\prime }}}{g_{sw}g_{sw^{\prime }}}\right]dwdw^{\prime }-\frac{1}{2}R\rho^{2}\iint g_{sw}g_{sw^{\prime }}\left[1-g_{ww}\right]dwdw^{\prime }\\ =-\sum_{pairs}\left[I_{ww}+S_{vol}\right] \end{array}.$$

*I*_*ww*_ is best understood as a mutual information (MI) term as it represents the additional correlation between two water molecules that is not captured by the solute-water entropy term. The *S*_*vol*_ term accounts for the exclusion of solvent from the volume occupied by other solvent molecules and is related to the Kirkwood-Buff integral (11,49). For a single water molecule, S_ww_ can be calculated by considering the pair correlation with all other water molecules in the system. In this work, we restrict the sum to pairs of water molecules within 4.0 Å. One can easily consider *I*_*ww*_ as a combination of two- and three-particle entropy terms:

(19) $$\begin{array}{c} I_{ww}=\frac{1}{2}R\rho^{2}\iint g_{sww^{\prime }}\;\ln\left[\frac{g_{sww^{\prime }}}{g_{sw}g_{sw^{\prime }}}\right]dwdw^{\prime }\\ =\frac{1}{2}R\rho^{2}\iint \left[g_{sww^{\prime }}\;\ln\;g_{sww^{\prime }}-g_{sww^{\prime }}\;\ln\;g_{sw}-g_{sww^{\prime }}\;\ln\;g_{sw^{\prime }}\right]dwdw^{\prime }\\ =S_{sww^{\prime }}-S_{sw}^{*}-S_{sw^{\prime }}^{*} \end{array},$$

where *S^∗^*_*sw*_ and *S^∗^*_*sw*'_ represent the two-particle entropies computed from the pair data and can be calculated using Eq. 14. The three-particle entropy (*S*_*sww*'_) can be calculated from the total distance (*d*_*pair*_) between two pairs of water molecules. In this case, both hydration sites are occupied in every snapshot for each doubly occupied cavity and thus all *n* frames contain pair data:

(20) $$\begin{array}{c} S_{sww^{\prime }}=R\left\{H_{abs}-H_{uni}\right\}\\ =R\left\{\frac{1}{n}\sum_{i=1}^{n}ln\left[\frac{nd_{pair}^{12}\pi^{6}}{\text{Γ}\left(7\right)}\right]-L_{0}+\gamma -\ln\left[\frac{64\pi^{4}}{\rho^{2}}\right]\right\}\\ =R\left\{\frac{1}{n}\sum_{i=1}^{n}\ln\left[\frac{nd_{pair}^{12}\pi^{6}}{720}\right]+\gamma -\frac{1}{n}\sum_{i=1}^{n}\ln\left[\frac{64\pi^{4}}{\rho^{2}}\right]\right\}\\ =R\left\{\frac{1}{n}\sum_{i=1}^{n}\ln\left[\frac{nd_{pair}^{12}\pi^{2}\rho^{2}}{46080}\right]+\gamma \right\} \end{array}$$

(21) $$d_{pair}=\sqrt{d_{total}^{2}+d_{total^{\prime }}^{2}}.$$

Г(7) is equal to 720. In practice, problems arise from combining KNN terms of different dimensionality in Eq. 19 (52). Thus, we use the method of permuted fill modes as described by Hensen et al. (53) The permuted set of distances (*d*_*perm*_) captures the correlation of the individual water molecules with the solute but decouples the correlation between the water molecules by computing the entropy of the artificially decorrelated data:

(22) $$\begin{array}{c} I_{ww}=S_{sww^{\prime }}-S_{sww^{\prime }}^{perm}\\ =R\left\{\frac{1}{n}\sum_{i=1}^{n}\ln\left[\frac{\rho^{2}\pi^{2}nd_{pair}^{12}}{46080}\right]+\gamma \right\}-R\left\{\frac{1}{n}\sum_{i=1}^{n}\ln\left[\frac{\rho^{2}\pi^{2}nd_{perm}^{12}}{46080}\right]+\gamma \right\}\\ =\frac{R}{n}\sum_{i=1}^{n}\ln\left[\frac{d_{pair}^{12}}{d_{perm}^{12}}\right] \end{array}.$$

Whereas the individual entropy terms, such as the one defined in Eq. 20, obey a power law convergence (as previously observed), the MI estimate in Eq. 22 does not appear to obey a power law convergence. It is also interesting to note that this MI estimate is not biased, as the bias terms from the two entropy estimates cancel one another. Within a singly occupied cavity, *I*_*ww*_ and *S*_*vol*_ are negligible because there are no significant water-water pair correlations. In a cavity with two or more water molecules, only pairs of subvolumes in which *g*_*sw*_ and *g*_*sw*'_ are nonzero will make a contribution to *S*_*vol*_. These regions are very small and *S*_*vol*_ is thus expected to be negligible. For this reason, we assume that *S*_*vol*_ is zero in all cases. As a comparison, the magnitude of *S*_*vol*_ in bulk water can be calculated from the radial distribution function and has a value of −0.99 cal/mol/K for the TIP4P-2005 water model, making a contribution of +0.29 kcal/mol to the excess free energy.

## Results and Discussion

We begin by considering the IFST estimates of enthalpy, entropy, and free energy contributions of each water molecule to the protein hydration free energy. The calculations for the 23 sites are presented in Table 2.

The standard deviations are all below 0.1 kcal/mol and generally much smaller. The predicted free energies are in the range from −2.6 to −16.0 kcal/mol, and this is in excellent agreement with previous IFST studies that showed a range from −1.9 to −17.2 kcal/mol (5,14). The predicted enthalpies are larger in magnitude than the entropies and make the dominant contributions to the free energies for all 23 water molecules. This is also in agreement with previous studies. The mean contribution of +1.82 kcal/mol is in good agreement with the contribution of +2.0 kcal/mol estimated by Dunitz, and the maximum contribution of +2.67 kcal/mol is not far above this value. As expected, the most favorable Δ*E*_*IFST*_ and unfavorable −*T*Δ*S*_*IFST*_ are found for protein cavities containing charged amino acid sidechains. Table 2 also shows that the *I*_*ww*_ term is small in magnitude for each pair of water molecules in the four doubly occupied cavities. The IFST and FEP results are reported in Table 3.

The agreement between IFST and FEP is extremely good, with an *R*^2^ coefficient of determination of 0.995 and a mean unsigned difference (MUD) of 0.45 kcal/mol. The accuracy of the estimates for *I*_*ww*_ are supported by the close agreement of Δ*G*_*IFST*_ and Δ*G*_*bind*_. In addition to analyzing the protein conformation from the crystal structure, we considered the effect of the protein conformation on the predictions of IFST in the case of T4 Lysozyme. This was achieved by analyzing 10 different protein conformations generated from a simulation with harmonically restrained heavy atoms. The results are presented in Table 4.

Comparing the results from the fixed crystal structure with the results from the harmonically restrained structure, the −*T*Δ*S*_*IFST*_ term is reduced from an average of 1.87 kcal/mol to an average of 0.90 kcal/mol, respectively. This is in agreement with previous work showing that the −*T*Δ*S*_*IFST*_ term is smaller in magnitude when using data from a harmonically restrained protein simulation and is the expected result, because of a blurring of the probability densities when the protein is mobile (18). The Δ*E*_*IFST*_ and Δ*G*_*IFST*_ terms also differ to a small extent, with the most notable difference being that the binding free energy is more favorable by 2.15 kcal/mol in the case of water 904. Comparing the IFST results from the fixed crystal structure with the results from the ensemble of 10 structures, the differences are lower than 1.5 kcal/mol in all cases, and the qualitative picture remains the same. However, it is worth noting that the standard deviations from the 10 simulations are relatively large, with a maximum of 1.40 kcal/mol for Δ*E*_*IFST*_ in the case of water 904.

## Conclusions

In this work, we have predicted the free energy of transferring water molecules from the bulk into a buried protein cavity using the methods of IFST and FEP. This can be viewed either as the binding free energy of the water molecule (Δ*G*_*bind*_) or the contribution of the water molecule to the hydration free energy of the protein (Δ*G*_*IFST*_). The free energy contributions are all strongly favorable and dominated by a favorable enthalpy component. The entropy contributions to the free energy are all unfavorable, but relatively small in magnitude. The water molecules with the strongest binding affinities tend to be in hydrophilic cavities making one (β-Lactamase W2073) or two (β-Lactamase W2327) hydrogen bonds with charged amino acids. Conversely, the water molecules with the weakest binding affinities tend to be in hydrophobic cavities (T4 Lysozyme W904) with very little potential for hydrogen bonding, as expected. The agreement between IFST and FEP for the 19 protein cavities in the five test systems is extremely good, with an *R*^2^ coefficient of determination of 0.995 and a MUD of 0.45 kcal/mol. To our knowledge, this study also represents the first calculation of the total two-particle entropy term (Δ*S*_*IFST*_) using information theory. The excellent agreement between IFST and FEP indicates that the mutual information terms between the pairs of water molecules (*I*_*ww*_) have been calculated correctly and that this term is negligible for water molecules in protein cavities. This is an important result because it demonstrates that the majority of the correlation is captured by the solute-water entropy (*S*_*sw*_) term. This means that the total change in water-water entropy (Δ*S*_*ww*_) is approximately equal to −*S*_*bulk*_ (rather than zero) and makes a significantly favorable contribution of −4.62 kcal/mol to the binding free energy for the TIP4P-2005 water model. This result is expected to hold for highly ordered water molecules in protein active sites. It is important to note that small values of *I*_*ww*_ in no way suggest an absence of correlation between the pair of water molecules in a doubly-occupied protein cavity. Solute-water and water-water correlations are explicitly wrapped up in the total two-particle entropy change (Δ*S*_*IFST*_), and the *I*_*ww*_ term serves to capture additional correlations not quantified by the *S*_*sw*_ term. One could equally well proceed by considering the water-water correlations first, but the choice of a solute reference frame significantly simplifies the calculations. In this study, we have considered cavities containing up to two water molecules. Studies on cavities with three or more water molecules would allow the accuracy of the two-particle approximation to be assessed. This is an important issue that should be addressed in future work. At the same time, it will also be important to estimate the *S*_*vol*_ term explicitly, as it may be significant and vary between buried and surface-exposed hydration sites.

The average prediction of −*T*Δ*S*_*IFST*_ is +1.82 kcal/mol and this is consistent with the work of Dunitz, who noted that water molecules in inorganic salts differ in entropy from bulk solvent by an amount corresponding to a free energy difference of approximately +2.0 kcal/mol. The largest value of −*T*Δ*S*_*IFST*_ in this study is found for a water molecule near two charged residues and is +2.67 kcal/mol. In comparison, the greatest difference in Dunitz’s analysis is +3.5 kcal/mol in the case of zinc sulfate monohydrate (10). It is important to note that the majority of entropy estimates in this work are for a fixed protein structure. The use of a fixed protein allows direct comparison of the IFST and FEP predictions and thus validation of the IFST calculations. It is clear that using a harmonically restrained or fully mobile protein structure within the current framework of IFST will lead to misestimation of the entropies because of a blurring of the probability densities. However, the results from calculations on 10 different fixed conformations of T4 Lysozyme suggest that IFST results should be robust in cases where the solute does not show a significant deviation in size, shape, or electrostatics. This is true for the cavities within these compact protein structures and should extend to many cases of interest in biology. Despite this, it is clear that further work is needed to develop probability-based statistical mechanical methods such as IFST to make accurate predictions.

The timescales for the IFST and FEP simulations are similar, though the timescales for FEP depend on the number of lambda windows. This makes FEP a technique that requires performing benchmark simulations for each particular case or user input in configuring the calculations. Conversely, the convergence of IFST calculations can be automatically monitored throughout a simulation until the required accuracy is reached. Importantly, a single IFST simulation is informative about every hydration site in a system whereas FEP requires a separate simulation for each water molecule. In addition, IFST can be used to study individual water molecules within a network whereas FEP is best suited to studying single water molecules or groups of water molecules as a whole. This is because annihilating a water molecule from a network requires artificially creating an unphysical vacuum in a hydration site. For this reason, IFST is unique in yielding spatially resolved prediction of water thermodynamics and this is extremely useful in identifying ligand-binding hotspots at protein surfaces (48) and guiding the design of high-affinity small-molecule inhibitors (47). The IFST calculation of the hydration entropy is defined in the context of a fixed solute. The use of a mobile protein has been shown to reduce Δ*S*_*IFST*_, and this can be attributed to a blurring of the probability densities. The effect of the protein conformation on the results of IFST has been investigated by performing simulations for 10 different fixed conformations of T4 Lysozyme. The results are very similar to those for the crystal structure conformation, with no difference greater than 1.5 kcal/mol. This suggests that IFST predictions will be robust if the protein confirmation does not deviate significantly from the conformation observed in the crystal structure. However, the standard deviations are significant and thus the choice of protein confirmation will affect the results if a single-protein confirmation is used. The method of combining results from multiple IFST calculations on an ensemble of protein confirmations allows one to account for molecular flexibility and estimate the coupling of solute and solvent degrees of freedom. Clearly, FEP has the major advantage of considering molecular flexibility and, alongside thermodynamic integration, remains the tool of choice for estimation of absolute and relative binding free energies. However, when studying water molecules, IFST has a number of unique advantages and these make it a very useful tool for understanding the role of hydration in the structure and function of biological systems.

In summary, to our knowledge, we have developed a new approach combining KNN and MI to calculate the total two-particle entropy in the context of IFST, and we have shown that the resulting predictions for the contribution of water molecules in protein cavities to the hydration free energy agree extremely well with equivalent predictions using FEP. The predicted entropy contributions to the free energy are in the range of +0.46 to +2.67 kcal/mol and this is in excellent agreement with historical estimates. In the future, it will be interesting to apply the entropy estimates developed in this work to extended water networks at protein surfaces and within protein binding sites. In these cases, the coupling of solute and solvent degrees of freedom is expected to be more significant and will need to be treated appropriately.

## Figures and Tables

**Table 1:** The five proteins considered in this study along with crystallographic data, the number of singly and doubly occupied internal cavities, the initial charge of the protein before adding counterions, and the residues altered during the preparation stage

Protein	IL-1β	T4 Lysozyme	FKBP-2	CA-II	β-Lactamase
PDB ID	2NVH	3DKE	2PBC	3GZ0	2P74
Protein chain	A	X	A	A	A
Resolution (Å)	1.53	1.25	1.8	1.26	0.88
Single cavities	2	2	1	5	5
Double cavities	2	1	1	0	0
Initial charge	0	+9	0	0	+1
Residues altered	Q14 Flip	H31 Protonate	H55 ε-Hydrogen	H4 Protonated	H112 Flip
	H30 Protonate	N68 Flip	Q 72 Flip	H10 ε-Hydrogen	Q31 Flip
	Q48 Flip	Q69 Flip		H15 ε-Hydrogen	Q141 Flip
	Q116 Flip	Q123 Flip		H17 ε-Hydrogen	Q206 Flip
	N119 Flip	N140 Flip		H36 ε-Hydrogen	Q254 Flip
	Q126 Flip	N144 Flip		H64 ε-Hydrogen	
	N129 Flip	N163 Flip		H119 ε-Hydrogen	
	Q149 Flip			N178 Flip	
				N253 Flip	

**Table 2:** The results of the IFST calculations for the 23 hydration sites, with the mean and standard deviation from 10 blocks reported

System	PDB water ID	E_sw_ (kcal/mol)	E_ww_ (kcal/mol)	ΔE_IFST_ (kcal/mol)	−TS_sw_ (kcal/mol)	−TS_ww_ (kcal/mol)	−TΔS_IFST_ (kcal/mol)	ΔG_IFST_ (kcal/mol)
IL-1β	202	−15.93 ± 0.03	−2.69 ± 0.01	−7.05 ± 0.03	6.35 ± 0.04	0.07 ± 0.02	1.80 ± 0.05	−5.25 ± 0.04
IL-1β	204	−16.73 ± 0.02	−2.72 ± 0.01	−7.88 ± 0.02	6.41 ± 0.03	0.07 ± 0.02	1.86 ± 0.04	−6.02 ± 0.03
IL-1β	203	−14.56 ± 0.05	−1.97 ± 0.02	−4.96 ± 0.04	6.63 ± 0.05	0.10 ± 0.03	2.11 ± 0.06	−2.85 ± 0.04
IL-1β	207	−13.75 ± 0.02	−1.99 ± 0.02	−4.16 ± 0.03	5.77 ± 0.03	0.10 ± 0.03	1.25 ± 0.03	−2.91 ± 0.03
IL-1β	200	−21.21 ± 0.02		−9.48 ± 0.02	6.53 ± 0.04		1.91 ± 0.04	−7.57 ± 0.03
IL-1β	209	−19.04 ± 0.03		−7.33 ± 0.03	5.08 ± 0.04		0.46 ± 0.04	−6.87 ± 0.02
T4 Lysozyme	902	−22.13 ± 0.02	−2.95 ± 0.01	−13.50 ± 0.02	6.72 ± 0.03	0.05 ± 0.02	2.16 ± 0.03	−11.34 ± 0.04
T4 Lysozyme	905	−18.67 ± 0.02	−2.97 ± 0.01	−10.06 ± 0.02	6.45 ± 0.03	0.05 ± 0.02	1.88 ± 0.03	−8.18 ± 0.03
T4 Lysozyme	904	−16.23 ± 0.03		−4.65 ± 0.03	6.27 ± 0.03		1.65 ± 0.03	−3.00 ± 0.01
T4 Lysozyme	920	−21.10 ± 0.02		−9.54 ± 0.02	6.40 ± 0.03		1.78 ± 0.03	−7.76 ± 0.01
FKBP-2	207	−17.96 ± 0.01	−2.37 ± 0.01	−8.76 ± 0.01	6.01 ± 0.03	0.08 ± 0.01	1.48 ± 0.03	−7.29 ± 0.02
FKBP-2	208	−19.54 ± 0.03	−2.44 ± 0.01	−10.40 ± 0.02	6.09 ± 0.05	0.08 ± 0.01	1.56 ± 0.06	−8.85 ± 0.05
FKBP-2	203	−26.49 ± 0.02		−14.91 ± 0.02	7.03 ± 0.04		2.41 ± 0.04	−12.50 ± 0.02
CA-II	2004	−20.77 ± 0.03		−9.47 ± 0.03	6.47 ± 0.03		1.85 ± 0.03	−7.62 ± 0.02
CA-II	2015	−27.52 ± 0.03		−15.92 ± 0.03	6.85 ± 0.04		2.23 ± 0.04	−13.70 ± 0.01
CA-II	2031	−22.40 ± 0.02		−11.09 ± 0.02	6.28 ± 0.02		1.66 ± 0.02	−9.44 ± 0.02
CA-II	2042	−24.11 ± 0.02		−12.56 ± 0.02	6.59 ± 0.03		1.97 ± 0.03	−10.58 ± 0.02
CA-II	2055	−17.80 ± 0.02		−6.20 ± 0.02	6.66 ± 0.03		2.04 ± 0.03	−4.17 ± 0.03
β-Lactamase	2023	−16.08 ± 0.03		−3.95 ± 0.03	6.02 ± 0.04		1.40 ± 0.04	−2.55 ± 0.02
β-Lactamase	2048	−27.94 ± 0.01		−16.33 ± 0.01	7.09 ± 0.03		2.47 ± 0.03	−13.87 ± 0.03
β-Lactamase	2073	−28.60 ± 0.03		−16.47 ± 0.03	6.52 ± 0.03		1.90 ± 0.03	−14.57 ± 0.02
β-Lactamase	2105	−24.70 ± 0.02		−13.08 ± 0.02	5.93 ± 0.05		1.31 ± 0.05	−11.77 ± 0.04
β-Lactamase	2327	−30.27 ± 0.02		−18.70 ± 0.02	7.29 ± 0.02		2.67 ± 0.02	−16.03 ± 0.01
Minimum				−18.70			0.46	−16.03
Maximum				−3.95			2.67	−2.55
Mean				−10.28			1.82	−8.46

**Table 3:** The results of the FEP and IFST calculations for the 19 cavities

System	PDB water IDs	ΔG_bind_ (kcal/mol)	ΔG_IFST_ (kcal/mol)	Signed difference (kcal/mol)	Unsigned difference (kcal/mol)
IL-1β	202 and 204	−11.77	−11.27	−0.49	0.49
IL-1β	203 and 207	−6.18	−5.76	−0.42	0.42
IL-1β	200	−7.09	−7.57	0.49	0.49
IL-1β	209	−6.90	−6.87	−0.03	0.03
T4 Lysozyme	902 and 905	−20.41	−19.52	−0.89	0.89
T4 Lysozyme	904	−3.33	−3.00	−0.33	0.33
T4 Lysozyme	920	−8.29	−7.76	−0.53	0.53
FKBP-2	207 and 208	−16.70	−16.13	−0.57	0.57
FKBP-2	203	−13.07	−12.50	−0.57	0.57
CA-II	2004	−8.21	−7.62	−0.59	0.59
CA-II	2015	−14.05	−13.70	−0.36	0.36
CA-II	2031	−10.06	−9.44	−0.62	0.62
CA-II	2042	−11.09	−10.58	−0.51	0.51
CA-II	2055	−4.29	−4.17	−0.12	0.12
β-Lactamase	2023	−2.31	−2.55	0.24	0.24
β-Lactamase	2048	−14.30	−13.87	−0.44	0.44
β-Lactamase	2073	−13.98	−14.57	0.59	0.59
β-Lactamase	2105	−12.06	−11.77	−0.29	0.29
β-Lactamase	2327	−16.53	−16.03	−0.51	0.51
Mean				−0.31	0.45

**Table 4:** The results of the IFST calculations for 10 conformations of T4 Lysozyme, alongside the results for the first 10 ns of the fixed crystal structure simulation, and the results for the 10 ns harmonically restrained simulation

PDB water ID	Fixed crystal structure			Ten simulation structures			Harmonically restrained		
	ΔE_IFST_ (kcal/mol)	−TΔS_IFST_ (kcal/mol)	ΔG_IFST_ (kcal/mol)	ΔE_IFST_ (kcal/mol)	−TΔS_IFST_ (kcal/mol)	ΔG_IFST_ (kcal/mol)	ΔE_IFST_ (kcal/mol)	−TΔS_IFST_ (kcal/mol)	ΔG_IFST_ (kcal/mol)
902	−13.50	2.16	−11.34	−13.08 ± 0.83	2.32 ± 0.32	−10.76 ± 0.84	−13.30	1.30	−12.00
905	−10.06	1.90	−8.16	−9.11 ± 0.39	2.12 ± 0.17	−6.99 ± 0.45	−8.80	1.38	−7.41
904	−4.65	1.66	−2.99	−5.62 ± 1.40	1.21 ± 0.25	−4.41 ± 1.26	−5.35	0.21	−5.14
920	−9.54	1.78	−7.76	−8.31 ± 0.96	1.53 ± 0.29	−6.79 ± 0.84	−8.52	0.70	−7.82

For the 10 conformations, the means and standard deviations are reported.
